# Imipridones ONC201/ONC206 + RT/TMZ triple (IRT) therapy reduces intracranial tumor burden, prolongs survival in orthotopic IDH-WT GBM mouse model, and suppresses MGMT

**DOI:** 10.18632/oncotarget.28707

**Published:** 2025-03-27

**Authors:** Lanlan Zhou, Leiqing Zhang, Jun Zhang, Laura Jinxuan Wu, Shengliang Zhang, Andrew George, Marina Hahn, Howard P. Safran, Clark C. Chen, Attila A. Seyhan, Eric T. Wong, Wafik S. El-Deiry

**Affiliations:** ^1^Laboratory of Translational Oncology and Experimental Cancer Therapeutics, Warren Alpert Medical School, Brown University, RI 02903, USA; ^2^Department of Pathology and Laboratory Medicine, Warren Alpert Medical School, Brown University, RI 02903, USA; ^3^Joint Program in Cancer Biology, Brown University Health System and Brown University, RI 02903, USA; ^4^Legorreta Cancer Center at Brown University, RI 02903, USA; ^5^Department of Medicine, Hematology/Oncology Division, Brown University Health System and Brown University, RI 02903, USA; ^6^Department of Neurosurgery, Brown University Health and Brown University, RI 02903, USA; ^7^Departments of Medicine, Radiation Oncology, Neurosurgery and Neurology, Brown University, RI 02903, USA

**Keywords:** glioblastoma multiforme, IDH, ONC201, ONC206, MGMT, temozolomide, radiotherapy

## Abstract

Glioblastoma remains a lethal brain tumor in adults with limited therapeutic options. TIC10/ONC201, a first-in-class imipridone we discovered, achieved meaningful therapeutic effects in phase I/II trials in patients with diffuse gliomas (DG’s) harboring H3K27M mutations, and currently the drug is in randomized phase III testing (ACTION trial; NCT05580562). ONC201 targets mitochondrial protease ClpP to disrupt oxidative phosphorylation and trigger the integrated stress response (ISR), TRAIL/DR5, and tumor cell death. While ONC201 and its analog ONC206 are undergoing clinical trials as single agents, there is limited information on their interactions with stand-of-care therapy. We show that ONC201 and ONC206 synergize with temozolomide (TMZ) and Radiotherapy (RT). ONC201 enhances TMZ- or RT-induced apoptosis, ISR and cytotoxicity. ClpP-silencing suppresses ONC201-induced cytotoxicity but not TMZ. Both ONC201 and ONC206 reduce expression of TMZ-resistance mediator MGMT observed in H3K27M-mutated DG cells following treatment with imipridones+TMZ. Cytokine profiling indicates distinct effects of ONC201 relative to TMZ treatment. These results suggest mechanisms underlying ONC201’s anti-tumoral activity are distinct from those associated with TMZ or RT with potential for synergy between these three treatments. Triple ONC201+RT+TMZ (IRT) therapy prolonged median survival to 123 days with tail on survival curve (3-of-7 mice alive beyond 200-days) in orthotopic U251 GBM model versus ONC201 (44-days; *p* = 0.000197), RT (63-days; *p* = 0.0012), TMZ (78-days; *p* = 0.0354), ONC201+RT (55-days; *p* = 0.0004), ONC201+TMZ (80-days; *p* = 0.0041) and RT+TMZ (103-days; *p* > 0.05). By 231-days, the only surviving mice were in IRT group. Our results support investigation of ONC201/ONC206 in combination with RT/TMZ (IRT) in GBM or H3K27M mutated DG therapy.

## INTRODUCTION

CNS tumors are among the most lethal cancers in children and adults. There are more than 13,000 new cases of malignant brain tumors diagnosed each year in the US alone. Glioblastoma (GBM) is the most common, fast growing, and aggressive primary malignant tumor in the CNS in adults [1, 2] carrying a poor prognosis, with a median survival of less than 15 months with only 10% of patients responding to standard of care [3]. GBM is a fast-growing and aggressive brain tumor that accounts for ~60–70% of malignant gliomas and ~15% of CNS tumors [4]. Moreover, diffuse intrinsic pontine glioma (DIPG), another lethal pediatric brainstem tumor primarily associated with the H3K27M mutation, displays a dire 5-year survival rate of less than 1%, primarily associated with the H3K27M mutation [5]. The standard of care (SOC) for GBM is maximally safe surgical resection, followed by concurrent radiation therapy (RT) and temozolomide (TMZ) for 6 weeks, then adjuvant TMZ for 6 months, yet the overall survival (OS) rates remain relatively low. Radiation is the primary treatment modality for DIPG but the benefit from concurrent temozolomide is questionable in light of higher systemic toxicities [6], while chemotherapies are offered to infants upfront to delay radiation until their brains become more developed.

TMZ, an alkylating agent, exerts its therapeutic action by methylating DNA adenine and guanine residues on single-stranded DNA [7]. The O-6-methyl-guanine (O-6-MeG) when methylated, if not repaired, can be mis-paired with thymine to trigger mismatch repair (MMR) [8]. If single- or double-stranded DNA breaks occur and are unrepaired, this ultimately results in growth arrest or apoptosis [9]. The presence or absence of O6-methylguanine-DNA methyltransferase (MGMT), an enzyme involved in DNA repair, diminishes or enhances TMZ’s efficacy in tumors with unmethylated and methylated promoter of this enzyme [7]. GBM with methylated MGMT promoter that inhibits MGMT protein expression has significantly longer OS compared to those that are unmethylated when treated with radiation and temozolomide [10].

ONC201 (also known as TIC10, originally discovered in the El-Deiry Lab [11, 12] as a TRAIL-inducing compound), is a novel first-in-class small molecule imipridone compound, that has shown promise in targeting various pathways involved in cancer progression, such as the extrinsic apoptotic pathway, upregulation of pro-apoptotic TRAIL receptor DR5 [13–15]. ONC201 was originally found to inactivate ERK/AKT leading to Foxo3a nuclear translocation and TRAIL gene activation [11]. Later it was discovered that ONC201 activates the integrated stress response (ISR) leading to ATF4-dependent upregulation of TRAIL receptor DR5 [16]. Other studies have documented depletion of cancer stem cells [17] and activation of the immune response mediated by natural killer cells that, in part, involve TRAIL/DR5 as a component of the host innate immune response against cancer [15]. Recent investigations have revealed that ONC201, along with its imipridone analogs ONC206 and ONC212, bind to mitochondrial ClpP as an agonist [18, 19], inhibiting oxidative phosphorylation as a component of its anti-cancer mechanism, eventually leading to apoptosis [12, 20]. Moreover, ONC201 and its analogs exhibit effectiveness against DIPG cell lines and cross the blood-brain-tumor barrier, suggesting their clinical applicability against CNS tumors.

Clinical studies and compassionate use cases of imipridone ONC201 have highlighted its promising efficacy, particularly in H3K27M-mutated tumors, including DIPG and GBM. Current trials exploring ONC201 as well as its analogs in combination with standard therapies seek to enhance their therapeutic potential in these aggressive tumors. Importantly, ONC201 is currently being explored in clinical studies in patients with various solid tumors and a randomized phase III study in patients with recurrent H3K27M-mutated diffuse glioma (ACTION trial; NCT05580562). ONC206 is currently being investigated in two Phase 1 studies in children and young adults with newly diagnosed or recurrent diffuse midline glioma (NCT04541082, NCT04732065). Notably, in recent compassionate use cases for ONC201, patients with H3K27M mutations in different brain tumors, particularly DIPG, glioblastomas, and other high-grade midline brain tumors have shown promising results, including significant tumor shrinkage and clinical improvements [21]. Furthermore, ONC201 and its analogs trigger ISR and this could attenuate the expression of DNA-damage repair proteins [22], which could potentiate the effects of radiation and concomitant TMZ for MGMT unmethylated GBM. The clinical experience with ONC201 in H3K27M–mutant pediatric DIPG provides further rationale for treatment with TMZ in combination with RT to investigate synergy in GBM and DIPG cells, *in vivo* models, and patients.

We investigated the synergistic effects of imipridone ONC201 in combination with RT and TMZ (IRT) as a triple combination therapy against GBM. Through *in vitro* and *in vivo* experiments on various brain tumor cell lines, including GBM, DIPG, and atypical teratoid rhabdoid tumors, we assessed cell viability, ISR activity, and apoptosis. Findings from *in vivo* orthotopic GBM mouse studies demonstrated that the triple combination treatment significantly prolongs survival and reduces tumor burden, decreases cell proliferation, and induces more apoptosis compared to single or dual therapies, indicating a promising avenue for GBM frontline treatment. We extended our studies and showed similar effects with ONC206 in IRT combination therapy. Importantly we observed unique cytokine profiles and suppression of MGMT expression in glioma cell lines by imipridones ONC201 and ONC206 as potential synergy mechanisms with TMZ. Suppression of MGMT protein was observed in H3K27M-mutated DIPG cell lines following treatment with ONC201 or ONC206 with or without TMZ. Overall, our preclinical findings support further exploration of the ONC201 and ONC206 IRT regimen as a potential treatment for GBM and diffuse gliomas with H3K27M mutations.

## RESULTS

### The combination of imipridone ONC201 and RT produces synergy with loss of cell viability in GBM cell lines

Previously, single-agent cytotoxicity of imipridone ONC201 or in combination with RT or TMZ was demonstrated *in vitro* [22–24]. We therefore hypothesized that ONC201 may synergize with both RT and TMZ in therapy of malignant brain tumors. To test this hypothesis, glioblastoma (GBM: SNB19, T98G and U251), diffuse intrinsic pontine glioma (DIPG: SF8628), and atypical teratoid rhabdoid tumor (ATRT: BT-12, BT-16) cell lines were tested using cell viability or colony formation assays with ONC201 up to 20 μM alone or in combination with radiotherapy up to 8 Gy or temozolomide up to 100 μM [24]. We used Western blot analysis to document drug-induced apoptosis of treated cells with single or combination therapy which showed synergy between ONC201 and RT and between ONC201 and TMZ with the best combination indices of 0.51 and 0.21 respectively [24].

**Figure 1:**
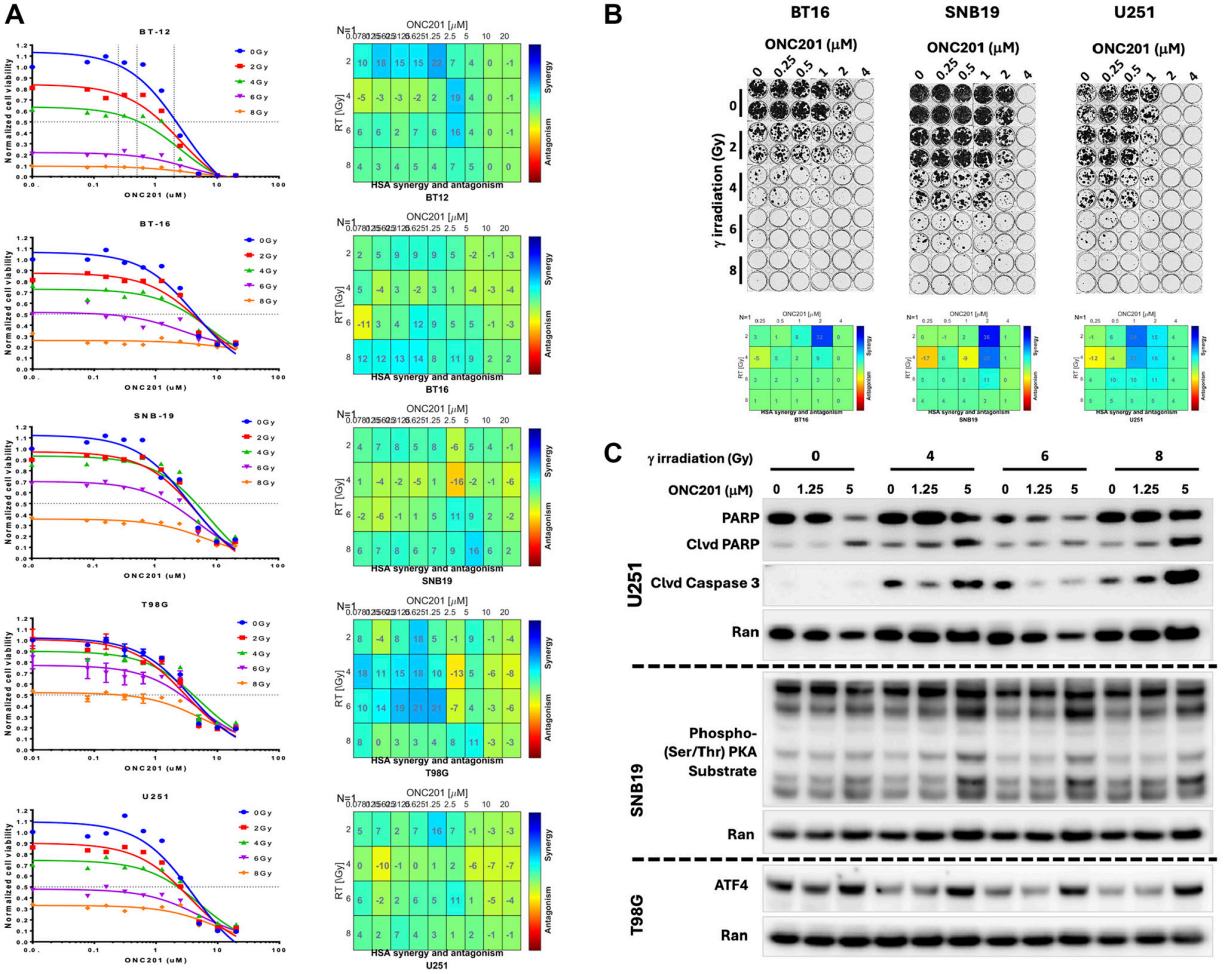
ONC201 synergizes with RT. (**A**) Combination of ONC201 and RT treated GBM cell lines SNB19, T98G, and U251, and atypical teratoid rhabdoid tumor (ATRT: BT-12, BT-16) cell lines show treatment effects on cell viability. (**B**) Colony formation assays with ONC201 up to 4 μM alone or in combination with radiotherapy up to 8 Gy. Blue boxes are dose combinations that show synergy between ONC201 and radiation therapy. Combenefit was used to evaluate the interaction of multiple treatments on short term cell viability and long-term colony formation assay - synergistic combinations are denoted by a synergy score greater than zero. (**C**) Western blots were utilized to assess induction of PKA substrate phosphorylation, induction of ATF4 as a marker of ISR activation, and multiple markers of cell death such as cleaved PARP and cleaved Caspase 3 in treated brain tumor cells.

We later investigated whether the combination of ONC201 and RT produced synergistic effects similar to what was reported previously [24]. To investigate the synergistic activity of ONC201 in combination with RT against brain tumor cell lines, we treated a panel of human brain tumor cell lines including GBM cell lines SNB19, T98G and U251, and atypical teratoid rhabdoid tumor (ATRT) cell lines BT-12 and BT-16 with varying doses of TRAIL pathway inducer ONC201 and RT. The cell viability after treatment demonstrated synergy between ONC201 and RT at various dose combinations in all cell lines tested (Figure 1A). The cell lines with the highest synergy were GBM cell lines SNB19, T98G and U251 cells as well as the ATRT BT12 cell line.

### The combination of ONC201 and RT synergistically suppresses colony formation by GBM cell lines

After observing synergistic cytotoxicity across GBM and ATRT cell lines in short-term cell viability assays, we explored whether similar sensitivity and synergy could be observed under longer-term exposure. A 7-day colony formation assay was conducted to assess the effects of ONC201 alone (up to 4 μM) or in combination with RT (up to 8 Gy) (Figure 1B). The LD90 was found under the exposure of ONC201 and radiation at 2 μM and 4 Gy for BT16, 2 μM and 6 Gy for SNB19, and 1 μM and 4 Gy for U251, respectively (Figure 1B). BT16 was the most sensitive followed by U251 and then SNB19 for reduction in colony-forming ability with combined ONC201 and RT treatment as compared to single agent treatment (Figure 1B). Thus, the long-term colony formation assay in Figure 1B demonstrates potent anti-cancer effects when ONC201 is combined with RT for the treatment of GBM and ATRT cells.

### ONC201 and RT activate ISR, enhance PARP and caspase cleavage and apoptosis

Having observed high levels of synergy across all tested GBM cell lines, we explored whether the treatment with ONC201 and RT induced apoptosis. T98G, SNB19 and U251 glioma cell lines were exposed to various doses of ONC201 and RT. Western blot analysis showed an ONC201 dose-dependent increase in the expression and activation levels of ATF4 protein, a mediator of ISR and a marker of ISR activation, possibly by specific eIF2 alpha kinases as described earlier [16]. Increased PKA substrate phosphorylation was also observed at the higher ONC201 dose level of 5 μM. Markers of apoptosis including cleaved PARP and cleaved caspase 3 were induced and reached the highest levels at 5 μM ONC201 and 8 Gy RT (Figure 1C). Overall, the data presented in Figure 1C supports the conclusion of synergy between ONC201 and radiation therapy in GBM cell lines.

### Imipridone ONC201 synergizes with temozolomide against GBM and DIPG cell lines

**Figure 2:**
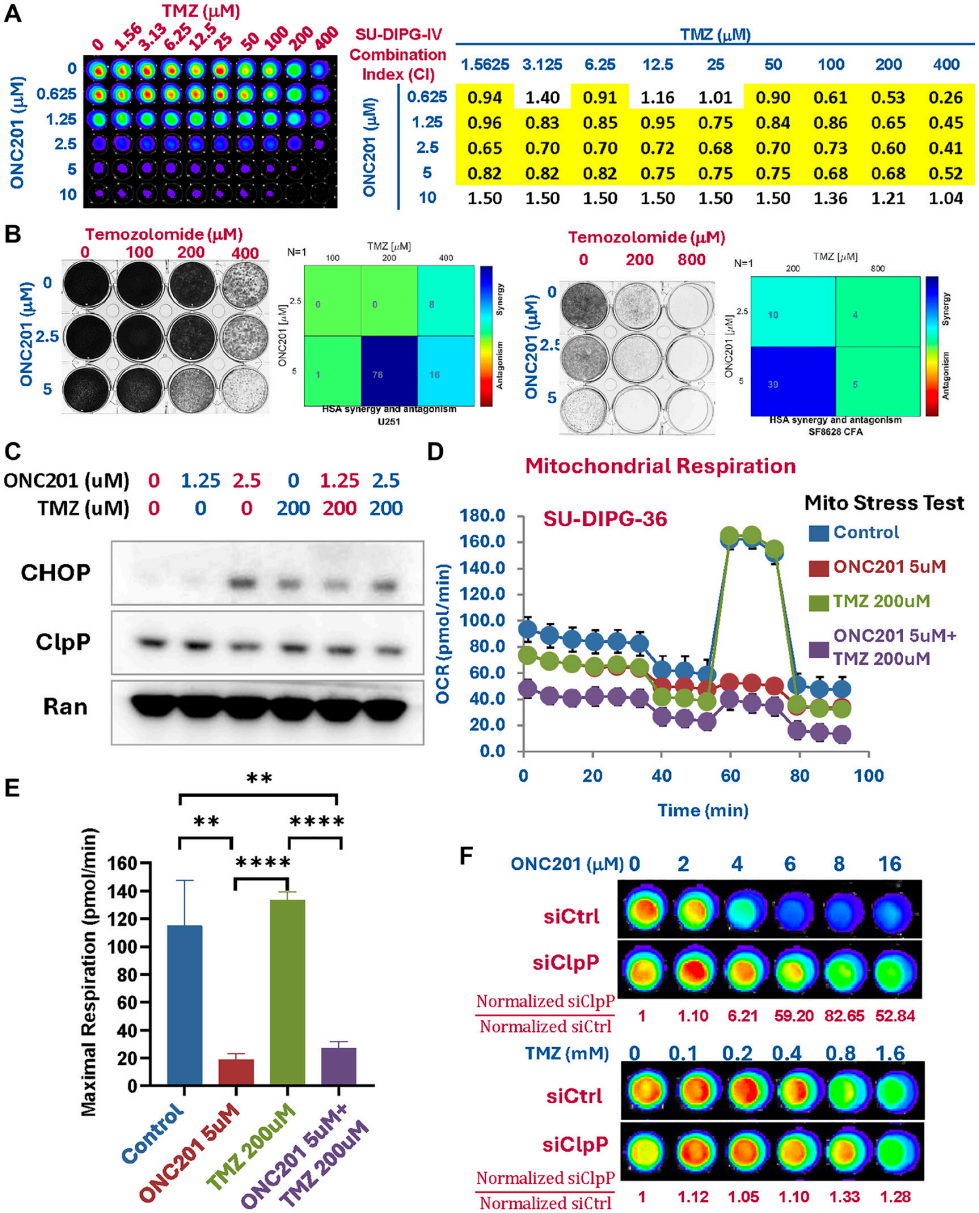
ONC201 synergizes with TMZ. (**A**) Cell viability assay diffuse intrinsic pontine glioma (DIPG) cell line SF8628 treated with the combination of ONC201 and TMZ. CellTiter Glo assays were performed to determine the cell viability after treatment. Combination indices (CI) were calculated by the method of Chou and Talalay using the CompuSyn software. Highlighted in boxes are dose combinations that show synergy. Data demonstrate synergy between ONC201 up to 5 μM and TMZ up to 400 μM. (**B**) Colony formation assays were performed to determine long-term cell viability after treatment. Combenefit was used to evaluate the interaction of two treatments on colony formation assay - synergistic combinations are denoted by a synergy score greater than zero. Data demonstrate the strongest synergy between 5 μM ONC201 and 200 μM TMZ. (**C**) Western blotting was utilized to assess induction of ATF4 and CHOP as markers of ISR activation, and cleaved PARP as a marker of cell death in treated brain tumor cells. The loading control in Figure 2C came from the same experiment and same gel for 0 Gy condition in Figure 3D. Data demonstrate synergy between ONC201 and TMZ. (**D**) Mitochondrial dysfunction induced by ONC201 is reflected in reduction of maximal cell respiration in the SU-DIPG 36 cell line. Treatment conditions shown in different colors as indicated. (**E**) Effect of ONC201 on oxygen consumption rate (OCR) tested in SU-DIPG 36 cell line using single-agent therapy with ONC201 or TMZ or doublet therapy using ONC201 in combination with TMZ. OCR of treated cells was measured by Seahorse Analyzer. Treatment conditions as in panel D. (**F**) siRNA knockdown of ClpP in GBM cell line U251 shows that ONC201 induced cytotoxicity depends on ClpP whereas siRNA knockdown of ClpP has no effect on TMZ-induced cytotoxicity.

Having observed single-agent cytotoxicity with monotherapy with ONC201 and TMZ [24], we investigated further whether the combination of ONC201 and TMZ produced synergistic effects. We performed both cell viability and colony formation assays to evaluate the combination of ONC201 and TMZ with the diffuse intrinsic pontine glioma (DIPG) cell line SF8628 and SU-DIPG-IV, and GBM cell line U251 using increasing concentrations of both drugs in combination. As shown in Figure 2A a Combination Index (CI) <1 was noted for cell viability assay across all concentrations of TMZ tested and ONC201 up to 5 μM with strong synergy CI = 0.26 for SU-DIPG-IV. Similar synergism was observed in colony formation assay with strong synergy at combination of 5 μM ONC201 and 200 μM TMZ for U251 and SF8628. (Figure 2B).

### Imipridone ONC201 in combination with TMZ induces ISR in GBM cells

**Figure 3:**
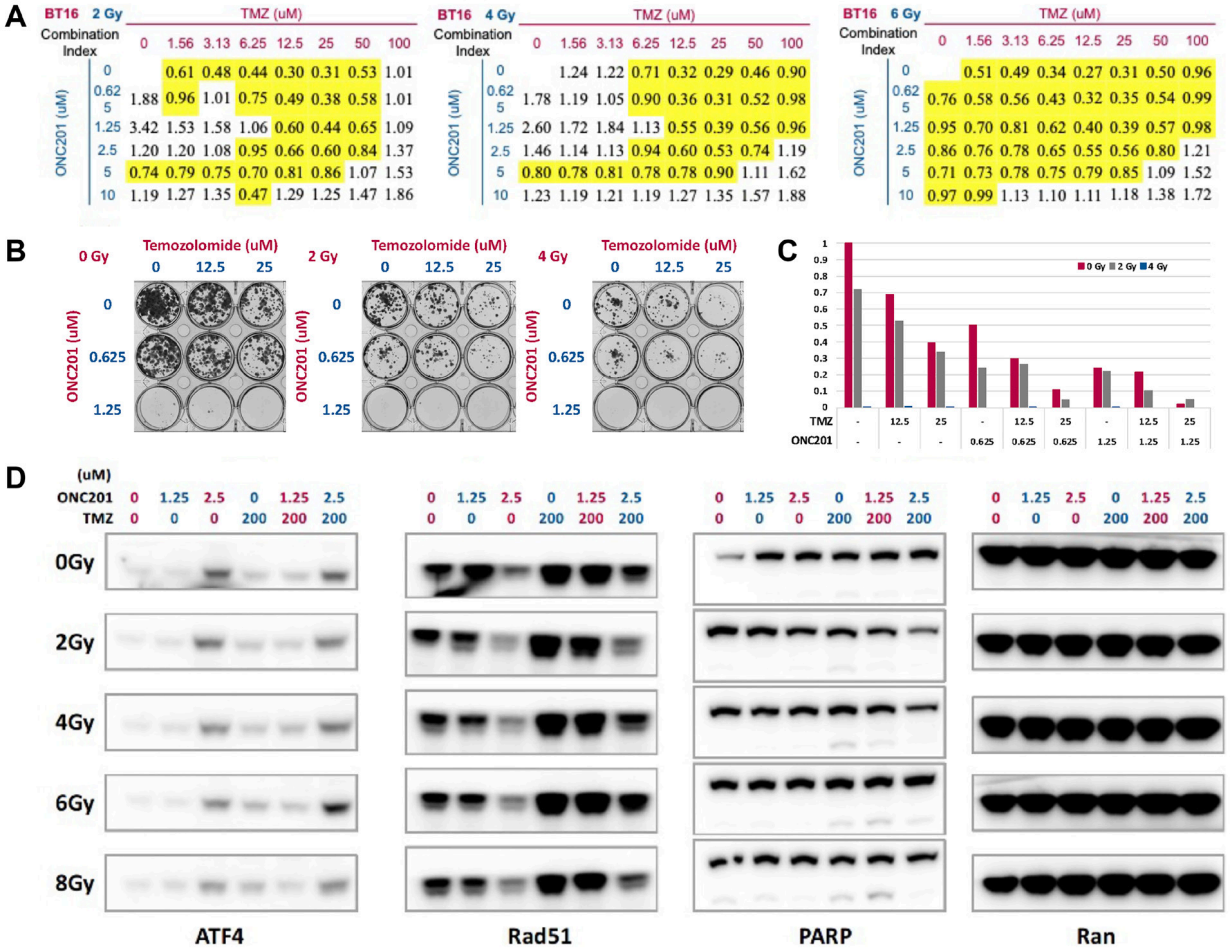
IRT triple combination of ONC201 with RT and TMZ synergizes in brain tumor cells. (**A**) IRT triple combination of ONC201, RT, and TMZ treated BT16 cells was assessed using cell viability. Combination indices are shown for rhabdoid tumor cell line BT16 treated at different radiation doses, TMZ and ONC201 as indicated. (**B**) Colony formation assays of GBM U251 cells treated with ONC201 alone or in combination with TMZ at indicated concentrations of each drug are shown. (**C**) Quantification of colony formation of GBM U251 cells after IRT triple combination treatment. Combination indices (CI) were calculated using the CompuSyn software with the lowest CI of 0.34 between 25 μM TMZ and 1.25 μM ONC201. (**D**) Western blotting to assess induction of ATF4 and CHOP as markers of ISR activation, cleaved PARP as marker of cell death and suppression of Rad51, a selective DNA repair target to radiosensitize glioma stem cells in treated GBM U251 cells. Data demonstrate synergy with IRT therapy with ONC201, RT, and TMZ.

**Figure 4:**
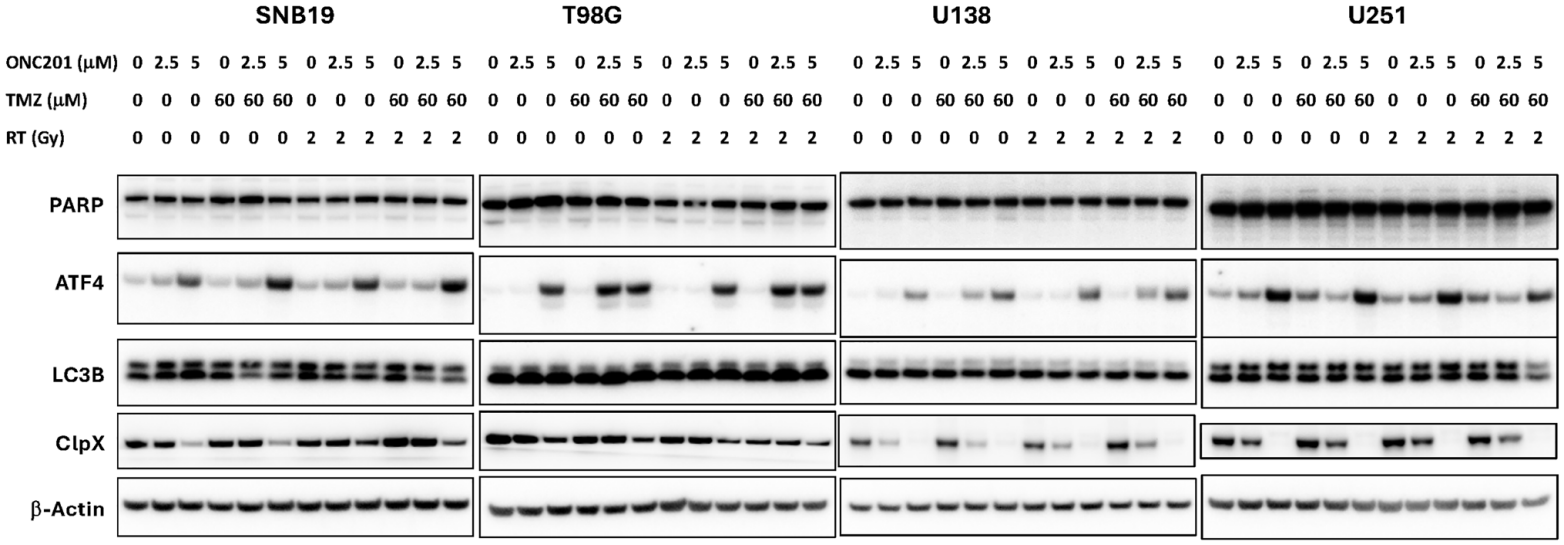
IRT triple combination of ONC201, TMZ and RT shows strong synergy, induces ATF as marker of ISR activation, and inhibits ClpX to unleash ClpP in SNB19, T98G, U138, and U251 GBM cells. IRT combination treatment effects with ONC201, RT, and TMZ on BT16, SNB19, and U251 GBM cell lines as assessed by Western blotting. IR combination treatment induces ATF4 and inhibited ClpX. Microtubule-associated protein light chain 3 (LC3) used as a specific marker to monitor autophagy shows no significant changes with treatments. Cells treated with ONC201, TMZ, and RT at indicated drug concentrations or radiation doses at 37^°C^ for 48 hours.

After observing significant levels of cytotoxic synergy in GBM cell lines, we investigated the potential activation of the ISR following treatment with ONC201 and TMZ. Western blot analysis showed that ATF4 and CHOP, markers of ISR activation and inducer of the apoptotic pathway, were upregulated upon treatment with ONC201 and/or TMZ (Figures 2C, 3D, 4). A robust synergy on ATF4 induction was observed when T98G and U138 were treated with 2.5 uM ONC201 and 60 uM TMZ - prominent ATF4 bands with combination treatment while no ATF4 bands with either single treatment. These findings suggest that ISR activation plays a significant role in synergy of ONC201 and TMZ.

### ClpP activation by imipridone ONC201 impairs oxidative phosphorylation and induces apoptosis following reduction of respiratory chain complex subunits

Based on findings of ISR activation and induction of apoptosis following treatment with ONC201 and TMZ, we then evaluated the relevance of mitochondrial protease ClpP (caseinolytic mitochondrial matrix peptidase proteolytic subunit that degrades damaged or misfolded proteins), a recently described binding and activation target of ONC201 [18, 19], on oxidative phosphorylation and mitochondrial function in SU-DIPG-36 cell line following treatment with ONC201 and TMZ. Our data demonstrate that treatment with ONC201 or ONC201 in combination with TMZ results in decreased basal and maximal oxygen consumption rate and that mitochondrial dysfunction induced by ONC201 is reflected in reduction of maximal respiration and spared respiration capacity in SU-DIPG-36 cell line (Figure 2D). Notably, TMZ alone has no effect on the maximal oxygen consumption rate in this cell line (Figure 2D, 2E).

### Imipridone ONC201-induced cytotoxicity depends on ClpP whereas TMZ-induced cytotoxicity is independent of ClpP

To further decipher the mechanism behind the decreased maximal respiration and spared respiratory capacity from ONC201 with or without TMZ, we investigated the dependency of mitochondrial protease ClpP in the GBM cell line U251. RNA interference studies were conducted using control versus ClpP siRNAs that were introduced into U251 cells, followed by treatment at concentrations of ONC201 and TMZ ranging from 0 to 16 μM and 0 to 1.6 mM, respectively. As expected, siClpP protected cells from loss of viability after ONC201 but not with TMZ treatment (Figure 2F). The result confirms that mitochondrial protease ClpP is an activation target of ONC201 and indicates that ONC201-induced cell death is probably mediated by mitochondrial dysfunction. By contrast, temozolomide induced cytotoxicity does not depend on ClpP.

### ONC201 synergizes with both RT and TMZ as IRT combination therapy in brain tumor cells

We hypothesized that ONC201 may synergize TMZ or RT in brain tumors. To test this hypothesis, we evaluated the combination of ONC201, RT and TMZ (named as ONC201 IRT therapy) with ATRT cell line BT16 and GBM cell line U251 using cell viability, colony formation, and Western blot assays. CellTiter Glo and colony formation and assays were performed to determine cytotoxicity effect after treatment with the IRT combination of ONC201, RT, and TMZ and found that triple combination exerts strong synergy in inhibiting cell viability of BT-16 (Figure 3A) and suppressing colony formation of U251 (Figure 3B, 3C). Collectively, ONC201 synergizes with TMZ and RT in cell viability (Figure 3A) and colony formation assays (Figure 3B, 3C) in brain tumor cells.

Western blots analysis was utilized to assess the apoptosis of treated cells and showed induction of ATF4 as marker of ISR activation, cleaved PARP as marker of cell death, and suppression of ATPase Rad51, a selective DNA repair target that radio-sensitizes glioma stem cells in brain tumor cells. Rad51 inhibition removes SOX2-expressing cells and abolishes clonogenicity [25] (Figure 3D). Single agent treatment with 2.5uM ONC201 induced ATF4 in GBM cell line U251. Adding 200 uM TMZ and 6 Gy RT increased ATF4 induction. GBM cell line U251 shows an increase in Rad51 expression after TMZ treatment alone. Rad51 levels are inversely correlated with radiosensitivity. Downregulation of Rad51 after ONC201 exposure markedly increases the cytotoxicity of TMZ and RT. These data demonstrate the synergism between ONC201, TMZ and RT, and this synergy is a result of induction of ISR, inhibition of homologous recombination (HR) and subsequent radiosensitization.

### IRT triple combination of ONC201, RT, and TMZ shows strong synergy in SNB19, T98G, U138 and U251 GBM cell lines, induction of both ISR and mitochondrial dysfunction

We sought to explore whether the synergy from IRT triple therapy that resulted in the induction of ISR and apoptosis is a generalizable phenomenon in other GBM cell lines. We therefore applied the combination of ONC201, TMZ and RT to SNB19, T98G, U138, and U251 glioblastoma cells and performed Western blot analysis of the expression of PARP, ATF4, LC3B and ClpX (Figure 4). Indeed, we found that ONC201 IRT triple combination induced ATF4 and inhibited ClpX, suggesting that ISR was activated and ClpP was most likely unleashed in all GBM cell lines tested. In addition, microtubule-associated protein light chain 3 (LC3), which serves as a specific marker for autophagy, had no significant changes with treatments. Collectively, our biomarker studies demonstrated that IRT triple combination treatment induces more ISR and inhibits ClpX to unleash mitochondrial ClpP (Figure 4).

### ONC201 or ONC206 IRT therapy shows synergies with RT and TMZ associated with distinct cytokine profiling

**Figure 5:**
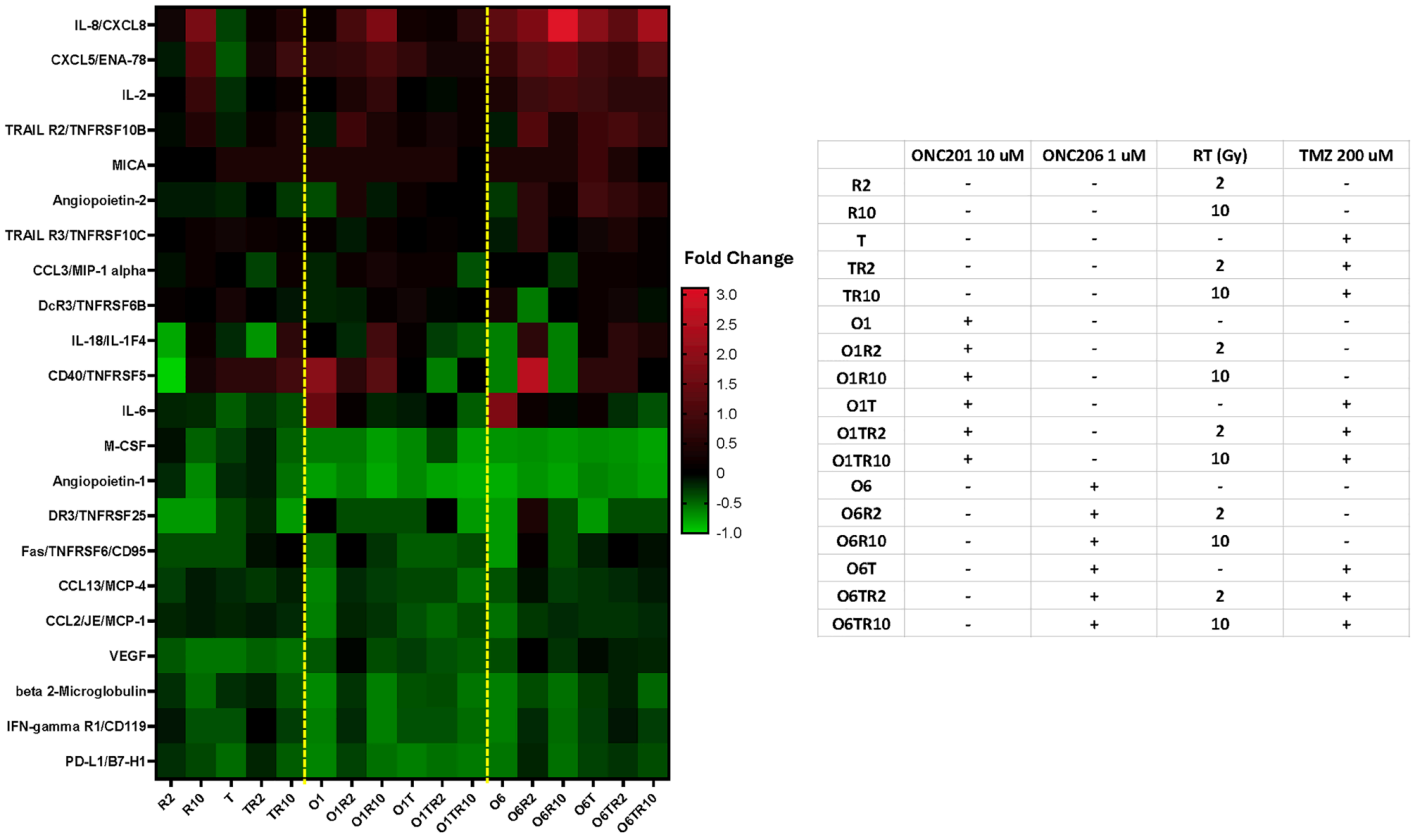
Differential cytokine profiling of IRT triple combinations of ONC201 or ONC206 with RT and TMZ for 48 hr. in U251 GBM cell line *in vitro*. U251 tumor cells treated with ONC201 or ONC206, TMZ, and RT at indicated concentrations or radiation doses for 48 hr. and cell culture supernatant analyzed with the Luminex 200. Fold-change is shown where red indicates a positive fold-change and green indicates a negative fold-change.

We treated GBM tumor cells (U251) with 10 μM ONC201 or 1 μM ONC206 as IRT therapy in combination with 200 μM TMZ and 2 or 10 Gy of radiation for 48 h and subsequently analyzed the cell culture supernatant using Luminex 200 technology (Figure 5). Several cytokines, chemokines, and growth factors associated with angiogenesis and/or EMT were found to be downregulated in the U251 cell line treated with either ONC201 or ONC206. Notably, angiopoietin-1 (a potent angiogenic growth factor that is critical for vessel maturation, adhesion, migration, and survival), M-CSF (a cytokine that regulates the proliferation, differentiation, and functional activation of monocytes), beta 2-microglobulin, PDL-1, IFN- γ R1, FAS, CCL2, CCL13, all had decreased secretion post-treatment with ONC201 in combination with TMZ and RT at both 2 and 10 Gy radiation doses. A similar but less pronounced decrease in these cytokines, chemokines, and growth factors was seen with IRT therapy using ONC206 in combination with TMZ and RT at both 2 and 10 Gy radiation doses. Likewise, several cytokines, chemokines, and growth factors associated with immunosuppression, including angiopoietin-1, Fas, and soluble PD-L1, were also downregulated post-treatment. Furthermore, ONC206 but not ONC201 in combination with TMZ and RT induced the secretion of TRAIL R3, CD40/TNFRSF5, CCL3/MIP-1a, IL-18/IL-1F4, GDF-15, CXCL5/ENA-78, IL-8/CXCL8, IL2, VEGF, angiopoietin-2 (an angiogenic growth factor that promotes cell death and disrupts vascularization), and TRAIL R2.

Two anti-cancer cytokines MICA and IL-2 are consistently increased with combinational treatment. The major histocompatibility complex (MHC) class I peptide A, MICA, is a ligand for NKG2D, a receptor on natural killer (NK) cells. MICA shedding by cancer cells causes effective escape from NKG2D recognition and allows the development of cancers. The soluble MICA protein can bind to NKG2D on NK cells and chronically inhibit them. Interleukin-2 (IL-2) stimulated the proliferation and activation of tumor infiltrating NK and T-cells.

Two pro-cancer cytokines M-CSF and Angiopoietin-1 are remarkably decreased with combinational treatment. Macrophage colony-stimulating factor (M-CSF) is a cytokine that’s elevated in glioblastoma (GBM) tissue and serum. M-CSF is a key regulator of macrophage survival, proliferation, and differentiation. It also plays a role in the immune response to tumor cells. Angiopoietin-1 (Ang1) is a growth factor that regulates tumor-induced angiogenesis in glioblastomas.

### ONC201 IRT in combination with RT and TMZ is an effective antitumor agent in mice with orthotopic GBM

Our cell culture studies above showed that IRT therapy with ONC201 synergizes with RT and TMZ in GBM cell lines SNB19, T98G, U138, and U251 and ATRT cell line BT16. IRT-treated cells induce ATF4 and CHOP as markers of ISR activation, cleaved PARP or cleaved caspase 3 as markers of apoptosis following IRT triple treatment regimen in cell culture experiments. We next sought to explore whether this IRT triple treatment also synergizes to induce ISR and apoptosis in an orthotopic GBM mouse model following treatment with ONC201 in combination with TMZ and RT.

**Figure 6:**
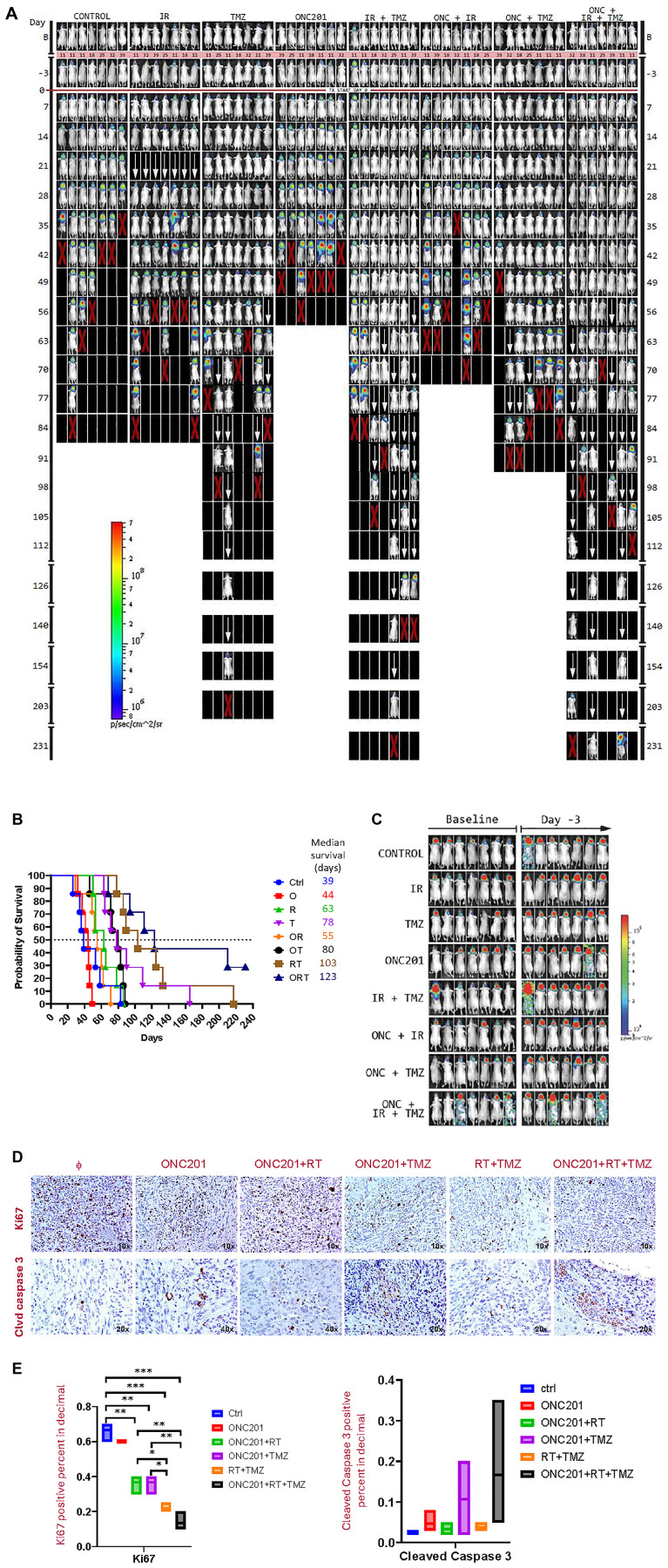
IRT triple combination of ONC201, RT, and TMZ for 4 weeks significantly prolongs mouse survival and reduces tumor burden in an orthotopic brain tumor model. (**A**) Randomized treatment group mice received weekly treatment of ONC201 (100 mg/kg p.o.) and/or radiotherapy (2 Gy local irradiation) and/or TMZ (20 mg/kg i.p.) for four weeks for long-term survival and tumor monitoring. (**B**) Long-term survival studies show that IRT triple combination of ONC201, RT, and TMZ significantly prolongs survival and reduces tumor burden as compared to single- and dual-treatments. (**C**) Tumors were growing at the time just before treatment was initiated: tumor images at baseline and day -3. (**D**) IHC assessment of IRT triple combination of ONC201, radiotherapy, and TMZ for 2 weeks shows reduced Ki67 expression and induction of cleaved caspase 3. (**E**) Quantification of short-term biomarkers demonstrating IRT triple combination treatment decreases tumor cell proliferation (Ki67) and induces more apoptosis (cleaved caspase 3).

Using intracranial injection of luciferase-expressing U251 GBM cells with a KOPF model 940 small animal stereotaxic frame and a Stoelting Quintessential Stereotaxic Injector, we confirmed tumor formation and growth using bioluminescence imaging. Findings from this study confirmed that tumors were growing at the time just before ONC201 IRT treatment was initiated by tumor images at baseline and day -3 (Figure 6C). Mice were randomized to receive weekly treatment of ONC201 (100 mg/kg p.o.) and/or TMZ (20 mg/kg i.p.) and/or RT (2 Gy local irradiation) for two weeks for short-term biomarker studies or four weeks for long-term survival and tumor monitoring (Table 1 and Figure 6A). In longer-term *in vivo* experiments, Kaplan Maier survival curve shows that, a four-week IRT triple therapy treatment with ONC201 in combination with TMZ and RT increases median survival of mice to 123 days as compared to double-agent or single-agent treatment regimens in an aggressive intracranial xenograft of human GBM U251-Luc cells (Figure 6A). Effectively, ONC201 cooperated with TMZ and RT to triple the survival duration of such brain tumor-bearing mice and reduce tumor size as compared to the control group (Figure 6A, 6B).

**Table 1:** Design of short and long-term IRT treatment *in vivo* studies

Design of *in vivo* studies			
Groups	4-week treatments for survival 2-week treatments for biomarkers		
	ONC201 100 mg/kg weekly, p.o.	TMZ 20 mg/kg weekly, i.p.	IR 2Gy weekly, local
Control	–	–	–
ONC201	+	–	–
TMZ	–	+	–
IR	–	–	+
ONC201+TMZ	+	+	–
TMZ+IR	–	+	+
ONC201+IR	+	–	+
ONC201+TMZ+IR	+	+	+

Thus, our longer-term survival studies show that the IRT triple combination of ONC201, radiotherapy, and TMZ significantly prolongs survival and reduces tumor burden as compared to single-treatment and dual-treatment combinations. Triple therapy with IRT prolonged median survival to 123 days with 3 of 7 mice alive beyond 200 days in an orthotopic U251 glioblastoma model relative to ONC201 (44 days; *p* = 0.000197), IR (63 days; *p* = 0.0012), TMZ (78 days; *p* = 0.0354), ONC201 + IR (55 days; *p* = 0.0004). ONC201 + TMZ (80 days; *p* = 0.0041) and IR + TMZ (103 days; *p* > 0.05) (Supplementary Figure 1). By 231 days, the only surviving mice were in the IRT group. These results support further larger *in vivo* studies, consideration for the incorporation of ONC201 or ONC206 into the current standard of care for glioblastoma in the upfront setting and also suggest potential synergies of triple therapy in tumors with H3K27M mutations that should be further explored in preclinical and clinical studies.

### An IRT triple combination of ONC201, RT, and TMZ for 2 weeks inhibits proliferation and induces apoptosis in an orthotopic brain tumor model

In addition to the long-term survival studies, we sought to explore a short-term cell proliferation (Ki67) and apoptosis markers in tumor specimens collected from mice two weeks after the start of a single, double, or IRT triple therapy with ONC201, RT, and/or TMZ. Short-term biomarker studies show that treatment with IRT triple combination of ONC201, TMZ, and RT for two weeks inhibited tumor cell proliferation and induced apoptosis assessed by measurements of Ki67 and cleaved caspase 3 respectively by immunohistochemical analysis of tumor specimens collected two weeks after the start of treatment (Figure 6D, 6E). Thus, these biomarker studies demonstrate that IRT triple combination treatment decreases tumor cell proliferation and induces more apoptosis compared to single-treatment or dual-treatment combinations.

### IRT triple combination therapy with ONC206, RT, and TMZ gives similar *in vivo* reduction in proliferation and induction of apoptosis as compared to ONC201 IRT therapy

**Figure 7:**
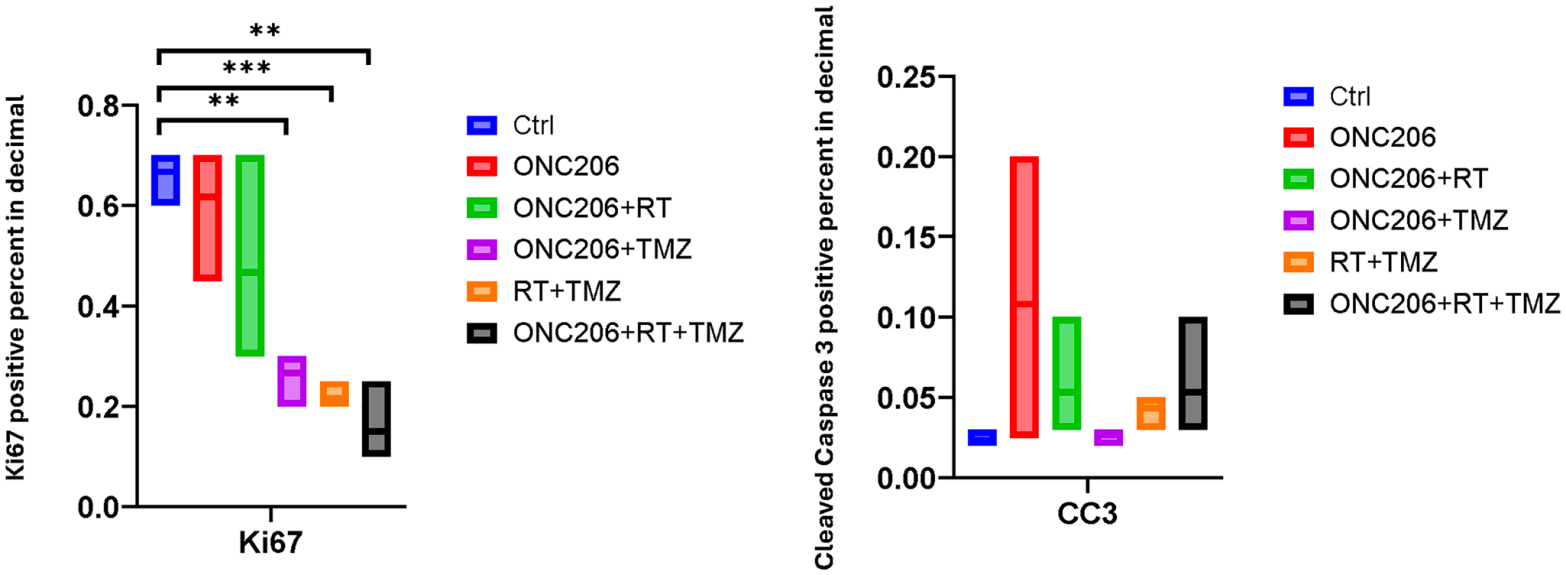
IRT triple combination of ONC206 with TMZ and RT for two weeks inhibits proliferation and induces apoptosis in the orthotopic U251 GBM model *in vivo*. A *g*roup of three mice received weekly treatment of ONC206 (100 mg/kg p.o.) and/or radiotherapy (2 Gy local irradiation) and/or TMZ (20 mg/kg i.p.) for two weeks for short-term biomarker studies. Short-term biomarker studies demonstrate that IRT triple combination of ONC206, TMZ, and RT for two weeks decreases tumor cell proliferation (Ki67) and induces apoptosis (cleaved Caspase 3). Quantification is shown from three mice per group.

Given that ONC206 has entered clinical trials for patients with brain tumors (NCT04732065, NCT04541082) we sought to gather preliminary data on its potential to synergize with TMZ and RT *in vivo* as we demonstrated above with ONC201. Mice that were orthotopically implanted with brain tumor cells (as in Figure 6) received weekly treatment of ONC206 (100 mg/kg p.o.) and/or TMZ (20 mg/kg i.p. weekly) and/or RT (2 Gy local irradiation) two weeks for short-term biomarker studies. The short-term biomarker studies (Figure 7) demonstrate that IRT triple combination of ONC206, TMZ, and RT for two weeks decreases tumor cell proliferation (Ki67) and induces apoptosis (cleaved Caspase 3). These results were similar to what we observed with the triple combination of ONC201, RT, and TMZ suggesting that both of the two imipridones ONC201 and ONC206 are worthy of further investigation in the clinic in combination with TMZ and RT.

### Reduction of expression of TMZ-resistance mediator MGMT in H3K27M-mutated DIPG cell lines following treatment with ONC201 or ONC206 with or without TMZ

**Figure 8:**
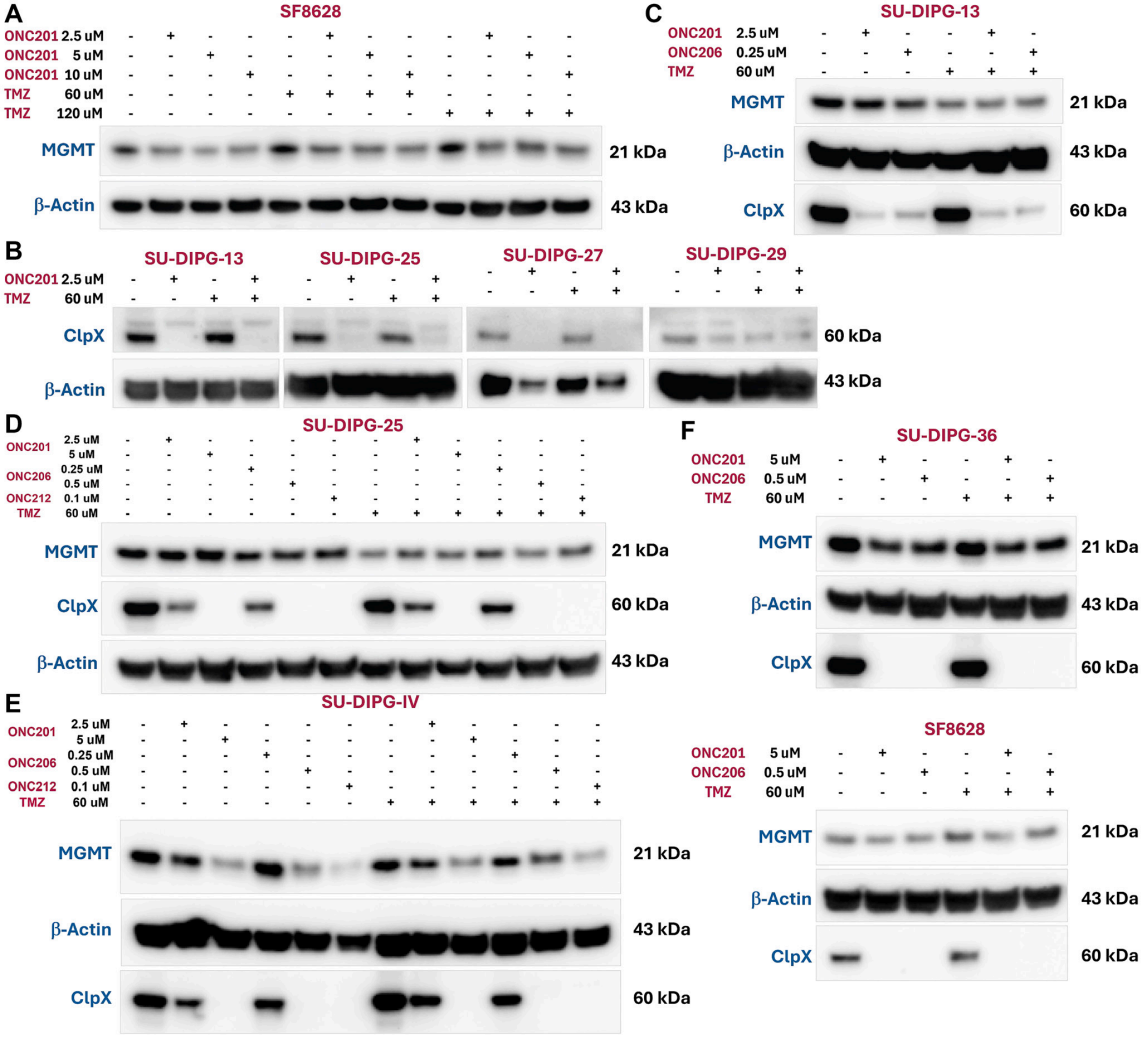
Imipridones (ONC201, ONC206 and ONC212) modulate MGMT and ClpX expression in DIPG cell lines. (**A**) ONC201 reduced MGMT expression in SF8628 cell line. (**B**) ONC201 inhibited ClpX expression in multiple DIPG cell lines (SU-DIPG-13, SU-DIPG-25, SU-DIPG-27 and SU-DIPG-29). Imipridones inhibited ClpX expression in a dose-dependent manner but did not reduce MGMT in SU-DIPG-13 (**C**) and SU-DIPG-25 (**D**) cell lines. (**E**) Imipridones decrease ClpX and MGMT expression in a dose-dependent manner in the SU-DIPG-IV cell line. (**F**) Imipridones (ONC201 and ONC206) inhibit ClpX expression and reduce MGMT in SU-DIPG-36 and SF8628 cell lines.

We recently reported that expression of MGMT in our commonly used GBM cell lines is low as compared to other solid tumor cell lines where it is quite high and that TMZ can have effects on tumor cell killing by T-cells within a few hours before there is time for cell division or mutations to occur [26]. MGMT promoter methylation leading to loss of MGMT protein expression is common in GBM and associated with response to TMZ [27, 28]. By contrast, MGMT promoter is rarely if ever methylated in H3K27M-mutated diffuse gliomas leading to TMZ resistance [29, 30]. We hypothesized that effects of imipridones on the ISR and global inhibition of protein translation might impact on MGMT expression and potential synergies when combined with TMZ for treatment of glioma cells. Because our GBM cell lines including the U251 cells used in our orthotopic brain tumor experiments have low or undetectable MGMT expression, we chose to test effects of imipridones on MGMT expression when used alone or in combination with TMZ in treatment of DIPG cells (Figure 8). We found evidence that imipridones (ONC201, ONC206 and ONC212) modulate MGMT and ClpX expression in DIPG cell lines. Both ONC201 and ONC206 reduced MGMT expression in SF8628 cells either in the absence or presence of TMZ (Figure 8A). We investigated the effects of the imipridones on ClpX and MGMT in additional DIPG cell lines including SU-DIPG-13, SU-DIPG-25, SU-DIPG-36, and SU-DIPG-IV (Figure 8B–8F). We found that to some extent there was a reduction of MGMT protein expression in the DIPG cell lines treated by the imipridones alone or in combination with TMZ. These novel findings suggest a potential mechanism of synergy between imipridones and TMZ through suppression of MGMT protein expression in DIPG cells that are typically unmethylated for MGMT promoter and resistant to effects of TMZ.

## DISCUSSION

Despite the utilization of multimodal therapies including RT and chemotherapy involving TMZ, the prognosis of GBM remains dismal, with consolidated treatment yielding a median survival of 14.6 months [31]. Furthermore, several randomized clinical trials investigating agents targeting EGFR, mTOR, and VEGF, along with multiple vaccine studies and immune checkpoint inhibitors, have yielded disappointing results after promising early-stage (phase I/II) studies in GBM. Our *in vitro* and *in vivo* findings demonstrate for the first time that IRT therapy combining ONC201 or ONC206 with TMZ and RT may be a safe and effective therapeutic strategy for GBM. Similar triple therapy using a putative TRAIL agonist TRA-8 in combination with TMZ and RT also resulted in enhanced cytotoxicity against glioma stem cells [32]. Their kinome analysis revealed that this enhanced cytotoxicity appears to be mediated by inhibition of JAK-STAT and Src tyrosine kinase signaling. WE have identified a novel mechanism for synergy between imipridones and TMZ involving suppression of MGMT expression that likely occurs through the imipridone-induced ISR with suppression of global protein translation. Our results and those of Arafat et al. [32] indicate that TRAIL is an important tumoricidal effector for glioblastoma. However, our results differ from those from Arafat et al. [32] by showing for the first time the effect on MGMT expression after imipridone treatment through the unique mechanism of imipridones that differs from TRAIL receptor agonists.

Our experimental findings from IRT triple combination treatment of GBM cell lines demonstrate that IRT triple combination treatment of ONC201 or its analog ONC206 in combination with TMZ and RT significantly inhibits cell survival, induces more apoptosis, disrupts mitochondrial function, inhibits various cytokines, chemokines, and growth factors associated with angiogenesis and/or EMT, immunosuppression or promotes factors associated with cell death and disrupts vascularization. Moreover, our *in vivo* studies using the orthotopic U251 GBM mouse model randomized to receive weekly IRT treatment of ONC201 or ONC206 and/or radiotherapy and/or TMZ for four weeks for either long-term survival and tumor monitoring or two weeks for short-term biomarker studies demonstrate that the IRT triple combination treatment significantly prolongs survival and reduces tumor burden compared to single and dual treatment combinations in the long-term studies. The IRT triple combination not only reduces tumor cell proliferation, but also induces more apoptosis, and inhibits ClpX to unleash mitochondrial ClpP in short-term biomarker studies. The effect of imipridones on MGMT in multiple DIPG cell lines provides a mechanism by which their combination with TMZ could overcome TMZ resistance in the clinic. This should be further tested across tumor types including H3K27M-mutated gliomas.

Our studies demonstrate that ONC201 synergizes with RT, induces apoptosis as indicated by the generation of multiple markers of cell death such as PARP, cleaved PARP, and cleaved Caspase 3, induces PKA substrate phosphorylation, upregulates ATF4 as a marker of ISR activation in treated brain tumor cells.

We further investigated the relevance of mitochondrial protease ClpP, a recently described binding target of ONC201 [18, 19], on oxidative phosphorylation and mitochondrial function in DIPG cells. Our findings reveal that mitochondrial dysfunction induced by ONC201 is reflected in reduction of maximal cell respiration in brain tumor cells. The observed mitochondrial protein degradation and cytotoxicity attributed to ONC201 is dependent on ClpP as knockdown of ClpP by siRNA protects multiple human cancer cell lines from ONC201-mediated cytotoxicity. Notably, temozolomide alone does not have an effect on the basal oxygen consumption rate. We demonstrated that IRT triple treatment of BT16 tumor cells with ONC201, TMZ and RT shows induction of ATF4 indicative of ISR activation, cleaved PARP as a marker of apoptosis, and suppression of RAD51, a selective DNA repair target to radiosensitize glioma stem cells in treated brain tumors. We observed that IRT triple combination treatment induces PKA substrate phosphorylation, induces ATF4/ISR activation, and inhibits ClpX to unleash ClpP, and multiple markers of cell death in treated in SNB19, T98G, U138, and U251 GBM cell lines.

Cytokine profiling from IRT-treated U251 GBM cells with ONC201 or ONC206 in combination with TMZ and RT for 48 hr. revealed that several cytokines, chemokines, and growth factors associated with angiogenesis and/or EMT were downregulated with both ONC201 and ONC206. Likewise, several cytokines, chemokines, and growth factors associated with immunosuppression were downregulated post-treatment, including angiopoietin-1, Fas, and soluble PD-L1. Interestingly, ONC206 but not ONC201 in combination with TMZ and RT induced the secretion of TRAIL R3, CD40/TNFRSF5, CCL3/MIP-1a, IL-18/IL-1F4, GD-15, CXCL5/ENA-78, IL-8/CXCL8, IL2, VEGF, angiopoietin-2, and TRAIL R2.

Long-term survival studies showed that IRT triple combination of ONC201, radiotherapy, and TMZ significantly prolongs survival and reduces tumor burden as compared to single-treatment and dual treatment combinations. We carefully documented that tumors were growing at the time just before IRT or other treatments were initiated by capturing tumor images at baseline and day -3. Evaluation of tumor images of surviving mice treated with indicated agents or a combination of agents on days 130 and 161 after implantation demonstrate that ONC201 synergizes with TMZ and RT and prolongs survival better than single or double treatments in the human GBM U251 orthotopic mouse model. Biomarker studies demonstrate that IRT triple combination of ONC201, RT, and TMZ for two weeks inhibits proliferation and induces apoptosis in tumor specimens. The biomarker studies demonstrate that IRT triple combination treatment also inhibits ClpX to unleash mitochondrial ClpP. The short-term *in vivo* IRT treatment study with ONC206 in combination with RT and TMZ achieves similar anti-tumor effects as evaluated by assessment of markers of cell proliferation (Ki67) and apoptosis (cleaved caspase 3) in tumor specimens collected two weeks after the start of treatment in the orthotopic U251 GBM model *in vivo*.

Based on our preclinical cell culture and *in vivo* data, IRT triple therapy with ONC201 or ONC206 synergizes with the TMZ and RT in an orthotopic glioma mouse model serves as a step towards clinical translation and the studies provide pharmacodynamic biomarkers.

Our findings demonstrate for the first time that IRT therapy combining ONC201 or ONC206 with RT and TMZ may be a safe and effective therapeutic strategy for GBM. Future studies may unravel novel signaling mechanisms following ONC201 or ONC206 plus TMZ and RT treatment of humanized and syngeneic immunocompetent *in vivo* model of GBM, and GBM patients. This includes the role of the ISR, TRAIL pathway, mitochondrial metabolism, ClpP, and MGMT as well as potential immune effects in the efficacy of the combination therapy. Furthermore, findings from this study might establish the IRT combination regimen of ONC201 or ONC206 in combination with TMZ and RT in tumors with or without H3K27M mutation and MGMT gene methylation [33] for targeted therapy.

Limitations include observed synergistic effects may be specific to certain cell lines, raising concerns about generalizability across different GBM tumor types including GBM tumor cells, organoids, and *in vivo* models with and without the H3K27M mutation and MGMT gene methylation. Mechanistic insights, while partially provided by Western blot analysis, require further studies such as transcriptomics or RNA-Seq to fully elucidate molecular pathways and consider potential off-target effects. Additional limitations include lack of use of syngeneic orthotopic mouse models and patient-derived xenograft humanized immunocompetent orthotopic GBM models. Lack of immune cell subset analysis, such as T-cell and NK-cell populations, and patient-derived models, highlights the need for validation across a broader spectrum of GBM subtypes and immune cell subtypes. The complexity of TME, including extracellular matrix, stromal cells, immune cells, fibroblasts, cytokines, tumor-associated myeloid cells/macrophages, T lymphocytes, B lymphocytes, Natural Killer cells, Neutrophils, Dendritic cells, GBM cells/Glioma stem cells among others [34] may significantly influence treatment responses.

While promising, additional research is necessary to confirm and translate these findings into clinical practice. Our data suggest synergy between the IRT triple therapy (i.e. imipridone ONC201 or ONC206, temozolomide and radiotherapy), including a tail on the survival curve not seen with mono- or dual-therapies. While we observed therapeutic benefit in mice with achievable dosing regimens that were not toxic, the findings support further exploration of mechanisms, PK, PD biomarkers, activity *in vivo*. Translation to the clinic that could be pursued in wildtype IDH1 GBM and H3K27M-mutated DIPG using combination of imipridones plus TMZ drugs at lower than recommended phase 2 doses with dose escalation with concurrent RT as the triple therapies have not been tested in humans.

It is noteworthy and clinically relevant that we observed synergy between ONC201 and TMZ in H3K27M-mutated SF8628 cells and have also previously reported TMZ effects on immune killing by T-cells of high MGMT-expressing tumor cells [26]. We showed that in SU-DIPG-36 cells that ONC201 suppresses oxygen consumption rate observed with TMZ alone, likely due to suppression of oxidative phosphorylation by ONC201. We also discovered the effect of imipridones on suppressing MGMT expression to potentially sensitize tumor cells with unmethylated MGMT promoter to the anti-tumor effects of TMZ. Of note, trials using ONC201 for H3K27M-mutated diffuse gliomas in the frontline setting exclude TMZ, and TMZ is generally not effective in these tumors due to unmethylated status of MGMT. Our results demonstrate, however, that in combination with TMZ the imipridones suppress MGMT expression suggesting potential benefit from combining imipridones with TMZ in CNS as well as other tumor types. Thus, the synergies and potentially beneficial therapeutic effects of combining ONC201 or other imipridones with TMZ, in addition to their combined use in the IRT combination could be further exploited. Our results support further studies combining ONC201 or ONC206 with RT and TMZ (IRT) in wildtype IDH1 glioblastoma, H3K27M mutated diffuse gliomas, or other tumors.

## MATERIALS AND METHODS

### Cell culture and reagents

Human brain tumor cell lines used in this study include GBM cell lines SNB19, T98G, U138 and U251 were obtained from the American Type Culture Collection (ATCC). Diffuse intrinsic pontine glioma (DIPG) cell line SF8628 was purchased from EMD Millipore. Six other DIPG cell lines (SU-DIPG-IV, SU-DIPG-13, SU-DIPG-25, SU-DIPG-27, SU-DIPG-29 and SU-DIPG-36) were a gift from Professor Michelle Monje (Stanford University). Atypical teratoid rhabdoid tumor (ATRT) cell lines BT-12 and BT-16 were kindly provided by Professor Michael Grotzer (University Children’s Hospital Zurich – Eleonorenstiftung). All cell lines were confirmed to be free of mycoplasma contamination using PCR testing methods. All GBM cell lines were cultured in their ATCC-recommended media supplemented with 10% fetal bovine serum (FBS) and 1% penicillin/streptomycin. ATRT and SF8628 were cultured in DMEM supplemented with 10% FBS and 1% penicillin/streptomycin. The six DIPG cell lines were cultured in tumor stem medium (basal medium plus growth factors). All cells are cultured at 37°C in a 95% humidified atmosphere containing 5% carbon dioxide. Chemotherapy agents used in the study were ONC201, ONC206 and ONC212 (obtained form Chimerix), solubilized in DMSO at a storage concentration of 20 mM; and TMZ was purchased from Selleckchem (Catalog No. S1237), reconstituted in DMSO at a storage concentration of 100 mM.

### Measurement of cell viability

Cells were seeded at a density of 3 × 10^3^ cells per well in a 96-well black plate (Greiner Bio-One, Monroe, NC, USA) and treated as desired after 24 hours. Cell viability was assessed using the CellTiter Glo assay (Promega, Madison, WI, USA). Cells were mixed with 25 μL of CellTiter-Glo reagents in 100 μL of culture volume, and bioluminescence imaging was measured using the Xenogen IVIS *in vivo* imaging system (Caliper Life Sciences, Waltham, MA, USA). The percentage of cell viability was calculated by normalizing the luminescence signal to control wells and was reported as % viability ± SD.

Dose-response curves were generated, and the half-maximal inhibitory concentration (IC50) was calculated using GraphPad Prism version 10.3.1 (RRID: SCR_002798). For IC50 determination, dose concentrations were log-transformed, and response data were normalized to the control, followed by a log (inhibitor) versus response (three parameters) analysis as described previously [35].

### Collection of cell supernatant samples for cytokine profiling

Cells were plated at 3.5 × 10^4^ cells in a 48-well plate (Thermo Fisher Scientific, Waltham, MA, USA) in complete medium and incubated at 37°C with 5% CO_2_. At 24 hours after plating, almost all the tumor cells were adherent to the bottom of the plate and the complete medium was removed and replaced with the drugged medium followed by RT. After 48 hours of treatment, cell culture medium was harvested and spun down at 1600 × g for 10 minutes. Supernatants were collected and frozen at −80°C until the measurement of cytokines, chemokines, and growth factors were performed. On the day of analysis, samples were thawed and centrifuged again.

### Cytokine profiling of drug-treated tumor cells

An R&D systems Human Premixed Multi-Analyte Kit (R&D Systems, Inc., Minneapolis, MN, USA) was run on a Luminex 200 Instrument (LX200-XPON-RUO, Luminex Corporation, Austin, TX, USA) per the manufacturer’s guidelines.

Sample levels of a custom panel of analytes (Figure 5) including Angiopoietin-1, Angiopoietin-2, M-CSF, beta-2-Microtubulin, PDL1/B7-H1, IFN-gamma, FAS, CCL2, CCL3, CCL13, DrC3, MICA, IL-6, CD40, IL18, GDF-15, CXCL5, IL-8, IL2, VEGF, TRAIL-R2, and TRAIL-R3 were measured for the drug treatment experiments. Analyte values were reported in picograms per milliliter (pg/mL). Fold change (treated – control)/control was presented as heat map using GraphPad Prism 10.3.1.

### Knockdown of ClpP gene expression by siRNA transient transfection

Cells were seeded in a 96-well plate and transfected transiently with 18 nM of control siRNA or siClpP using lipofectamine RNAi MAX (Invitrogen) following the manufacturer’s instructions and cells were cultured for 48 hours. After 48 hours, cell culture media was replaced with fresh media and treated with ONC201 or TMZ at indicated concentrations and further cultured for an additional 72 hours before CTG readout.

### Western blot analysis

Cells were lysed in RIPA buffer (Sigma-Aldrich, St. Louis, MO, USA) containing cocktail protease inhibitors (Roche, Basel, Switzerland). Equal amounts of cell lysates were electrophoresed through 4–12% SDS-PAGE and then transferred to PVDF membranes. The transferred PVDF membranes were blocked with 5% skim milk at room temperature, then incubated with primary antibodies indicated in a blocking buffer at 4°C overnight. Antibody binding was detected on PVDF with appropriate HRP-conjugated secondary antibodies by a Syngene imaging system (Syngene, Bangalore, India). The antibodies that were used in the manuscript are listed after the immunohistochemistry section.

### Colony formation assays

Approximately 200 brain tumor cells per well were cultured in 12-well plates. Cells were treated with different drugs or with radiation therapy at various doses. The cell colonies were fixed with methanol and stained with 0.1% Coomassie Blue at the end of the experiments. Stained plates were imaged with the Syngene imaging system and colonies were quantified with ImageJ Software (NIH).

### *In vivo* experiments

The experimental protocol for the *in vivo* study (IACUC Protocol # 22-02-0004) was approved by the Institutional Animal Care and Use Committee of Brown University (Providence, RI, USA).

#### Intracranial injection

With a KOPF model 940 small animal stereotaxic frame and a Stoelting Quintessential Stereotaxic Injector, NCRNU-Female, 5 weeks of age athymic nude mice (Taconics, https://www.taconic.com/mouse-model/ncr-nude) were used to inject 2 × 10^5^ U251-Luc expressing cells in 5 μL PBS orthotopically into the frontal region of the cerebral cortex a site 2.5 mm lateral (right), 1.5 mm anterior, and 2.5 mm ventral with respect to the bregma to produce a GBM tumor.

#### Treatment regimen

ONC201 was prepared at 25 mg/mL concentration in 10% DMSO + 20% Cremophor EL + 70% PBS. ONC206 was prepared at 25 mg/mL concentration in sterile water. TMZ was prepared at 2 mg/mL in sterile PBS. Local radiotherapy was delivered by Orthovoltage X-ray machine (Phillips Medical Systems RT 250 Therapy X-ray) with normal body parts shielded.

Cohorts of mice (*n* = 7) were treated with vehicle control, single agent with ONC201 or ONC206 (100 mg/kg weekly, p.o.), TMZ (20 mg/kg weekly, i.p.), RT (2Gy weekly, local), as well as with dual therapy with ONC201 or ONC206 + TMZ, ONC201 or ONC206 + RT, TMZ + RT, and triple therapy with ONC201 + TMZ + RT. All mice were imaged using the IVIS^®^ *in vivo* imaging system for bioluminescence after tumor cell injection to monitor tumor growth. All mice were followed daily to record survival. Overall survival was analyzed by plotting the survival data on a Kaplan-Maier survival curve.

### Immunohistochemistry

Excised tumor tissue samples were fixed using 10% neutral buffered formalin and embedded in paraffin. Tissue sections of approximately 5 µm were cut with a microtome and mounted on glass microscope slides for subsequent staining.

Hematoxylin and eosin (H&E) staining was performed for all tumor tissue specimens. Paraffin embedding and sectioning of slides and H&E staining were performed by the Brown University Molecular Pathology Core Facility. Slides were dewaxed in xylene and subsequently hydrated in ethanol at decreasing concentrations. Antigen retrieval was carried out by boiling the slides in 2.1 g citric acid (pH 6) for 10 min. Endogenous peroxidases were quenched by incubating the slides in 3% hydrogen peroxide for 5 min. After nuclear membrane permeabilization with Tris-buffered saline plus 0.1% Tween 20, slides were blocked with horse serum (Cat# MP-7401-15, Vector Laboratories, Burlingame, CA, USA), and incubated with primary antibodies overnight in a humidified chamber at 4°C. After washing with PBS, a secondary antibody (Cat# MP-7401-15 or MP-7402, Vector Laboratories, Burlingame, CA, USA) was added for 30 min, followed by diaminobenzidine application (Cat# NC9276270, Thermo Fisher Scientific, Waltham, MA, USA) according to the manufacturer’s protocol. Samples were counterstained with hematoxylin, rinsed with tap water, dehydrated in an increasing gradient of ethanol, cleared with xylene, and mounted with Cytoseal mounting medium (Thermo Fisher Scientific, catalog no. 8312-4). Slides were scanned with an Olympus VS200 slide scanner. All slides were reviewed and reported by an experienced pathologist.

**Table:** 

Antibody	Source	Catalog #	Concentration
PARP	Cell Signaling	9542	1:1000
cleaved PARP	Cell Signaling	9546	1:2000
Rad51	Santa Cruz	sc-8349	1:500
Ran	BD	610341	1:10000
pPKA substrate	Cell Signaling	9621	1:1000
ATF4	Cell Signaling	11815	1:1000
Ki-67	Cell Signaling	9449	1:800
Cleaved Caspase-3 (Asp175) Antibody	Cell Signaling	9661	1:400
CHOP	Cell Signaling	2895	1:1000

### MGMT and ClpX expression analysis after imipridone with or without TMZ in DIPG cells

DIPG cells were treated at indicated doses of imipridones and/or TMZ for 48 hours in Figure 8 before harvested for western blotting analysis. Antibodies for MGMT were obtained from Cell Signaling Technology cat #2739 and used at a titer of 1:1000 while Clpx antibody was obtained from Abcam cat #ab168338 and used at a 1:1000 dilution. β-Actin loading control antibody was obtained from Sigma-Aldrich cat #A5441 and used at a 1:5000 dilution.

### Statistical analysis

Combination Index (CI) was calculated using CompuSyn software to evaluate the interaction of multiple treatments - synergy is indicated when CI <1, additive CI = 1 and antagonism CI >1 [36]. The synergy score was calculated using Combenefit software to assess if there was a synergistic, additive or antagonistic relationship between two compounds. If the effect of a drug combination is greater than the additive effect, the synergy score is >0; if the effect of a drug combination is less than the additive effect, the synergy score is <0. A higher synergy score denotes greater synergy of the corresponding drug combination [37]. Although the combination effect analysis results are consistent using both software (data not shown), CompuSyn can analyze more than two treatment combinations but only manual data input is available, while Combenefit can copy/paste excel file to input data efficiently but can only analyze two treatment combinations.

GraphPad Prism (RRID: SCR_002798) version 10.3.1 was used for statistical analyses and graphical representation (GraphPad, San Diego, CA, USA). Data are presented as means ± standard deviation (SD) or standard error of the mean (SEM). The relations between groups were compared using two-tailed, paired Student’s *T*-tests or one-way ANOVA tests. Survival was analyzed with the Kaplan-Meier method and was compared with the log-rank test. For Kaplan Meier curves, *p*-values were generated using a Survival Difference Calculator (log rank test) with default parameter *ρ* = 0 (https://astatsa.com/LogRankTest/).

## SUPPLEMENTARY MATERIALS


