# Supplementary material for: Imipridones ONC201/ONC206 + RT/TMZ triple (IRT) therapy reduces intracranial tumor burden, prolongs survival in orthotopic IDH-WT GBM mouse model, and suppresses MGMT
Source: Oncotarget. 2025 Mar 27;16:230–48. doi: 10.18632/oncotarget.28707 (PMC11948724; doi:10.18632/oncotarget.28707)
Supplement: Supplementary file 1 [file oncotarget-16-28707-s001.pdf]

Imipridones ONC201/ONC206 + RT/TMZ triple (IRT) therapy reduces intracranial tumor burden, prolongs survival in orthotopic IDH-WT GBM mouse model, and suppresses MGMT

SUPPLEMENTARY MATERIALS

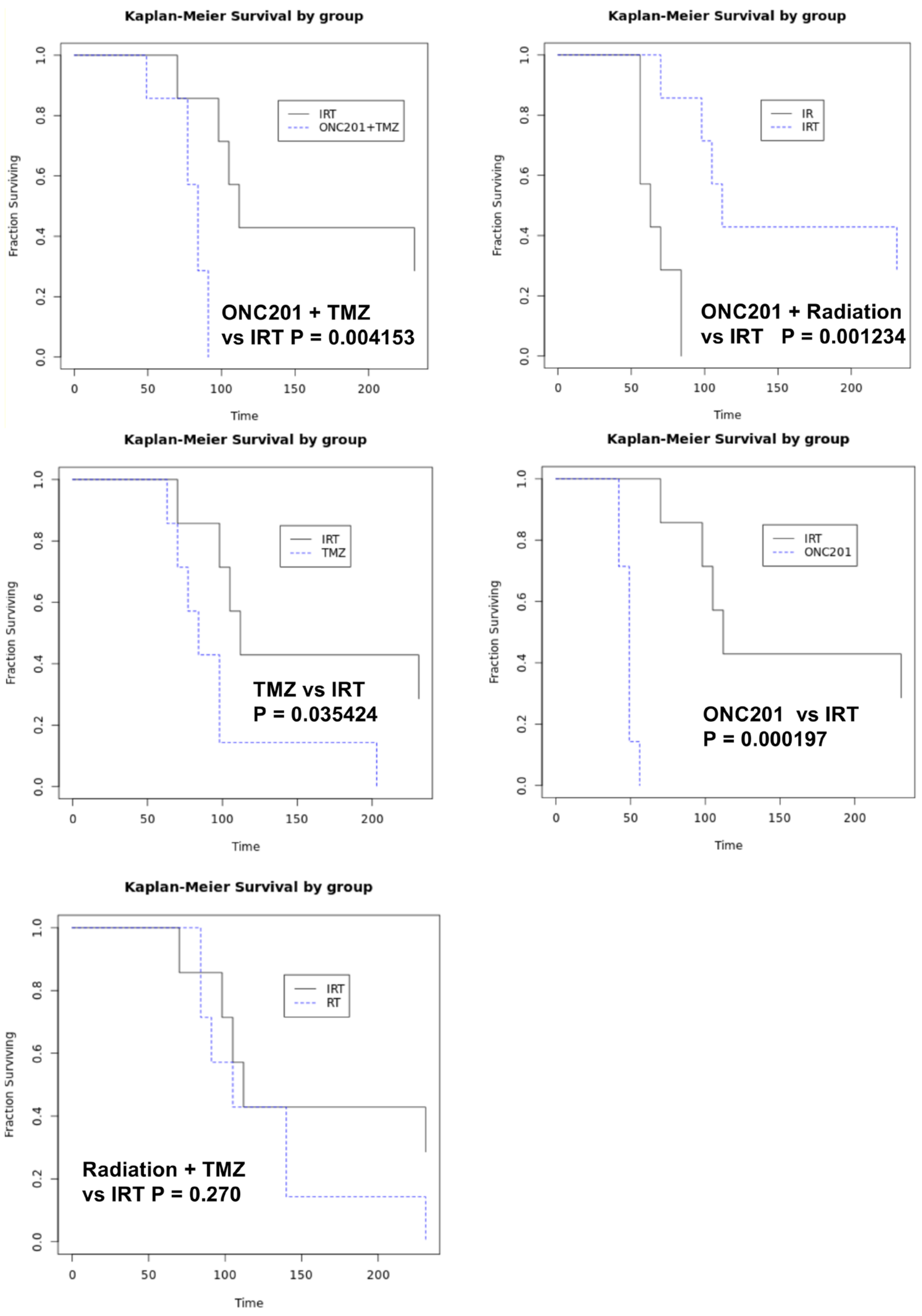

Supplementary Figure 1: Statistical analysis of mouse survival. Survival was analyzed with the Kaplan-Meier method and was compared with the log-rank test. For Kaplan Meier curves, pvalues were generated using a Survival Difference Calculator (log rank test) with default parameter $\rho = 0$ (https://astatsa.com/LogRankTest/). Survival of treatment cohorts with mono- or doublet therapy is compared to triple IRT therapy with *p*-values as indicated.
